# A Study of Piano Timbre Teaching in the Context of Artificial Intelligence Interaction

**DOI:** 10.1155/2021/4920250

**Published:** 2021-11-27

**Authors:** Cui Wei

**Affiliations:** Academy of Music, Hebei Normal University, Shijiazhuang, Hebei 050000, China

## Abstract

This paper provides an in-depth analysis and research on piano timbre teaching in the context of artificial intelligence interaction, a bold vision of piano teaching, proposes a feasible solution in terms of teaching modules in intelligent piano teaching for senior teachers, and proposes an implementation path for the integration of intelligent piano and piano teaching from the four main blocks of piano teaching. Based on the multiplicative harmonic model of monophonic signal, combined with the variability of timbre characteristics, an audio synthesis model with editable timbre is proposed, and the experimental results show that editing the timbre parameters in the model can realize timbre modification, and the synthesized timbre conforms to the piano timbre characteristics. Based on the timbre analysis and the timbre synthesis model, a piano timbre library generation system is designed. The detailed design of the software modules such as audio file reading and writing, audio information analysis, timbre parameter acquisition, timbre synthesis, and simulated performance is given. The system can generate piano timbre libraries of different qualities flexibly and meet the requirements of timbre realism. The teaching experiment designed for teaching practice from solo teaching, and the practice target is first-year undergraduate students in the university, and the practice period is six weeks, and finally, the feasibility of intelligent piano teaching application is analysed by combining the experimental results. Through the teaching objectives, teaching content, and teaching methods, teaching environment reflects intelligent piano teaching to make up for the limitations of traditional piano teaching. Analyse the development trend of intelligent piano teaching in the context of artificial intelligence interaction, and explore the value of intelligent piano teaching.

## 1. Introduction

With the continuous development of contemporary art, the combination of musical art expression and modern computer technology is becoming increasingly widely used. Analysing the situation of major international musical instrument exhibitions in recent years, the musical instrument manufacturing industry in developed countries has started the research and development of intelligent pianos and has made great progress. Some internationally renowned piano brands have started to display sample pianos equipped with modern technological functions, which gradually attract the attention of the international music and musical instrument industry [1]. The imported audio file needs to be parsed to obtain audio data information. Then, the audio data is frequency spectrum transformed, and the timbre characteristic information is analysed; the timbre parameters are obtained through the human-computer interaction interface, and the piano timbre is generated. Play the generated timbre in simulation, if the timbre effect is satisfactory. Some experts predict that intelligent pianos will be the direction of development in the piano industry. For digital pianos and smart pianos, the sound source is an important factor that affects their quality and grade. An intelligent piano with high-quality sound sources and characteristics can form good and continuous competitiveness in the market [2]. The intelligent digital piano series launched by Helen Piano has very high requirements for the sound source, taking the Helen Intelligent Piano Angel Series DUAII model as an example, it adopts a dual sound source system, a high-quality digital electroacoustic sound source, and a high-quality soundboard physical sound source, which is the same as the traditional piano sound, pure sound collection of 9 kinds of famous European piano quality tones, up to 7 layers of tone performance, 128 realistic tones [3]. With the tone synthesis system to simulate the real tone of the instrument and the flexibility to modify and create new tones by editing the tone, it can enable smart and digital instruments to obtain a richer expression of the instrument. In the field of art, visual art and auditory art are the most common forms of art, each of which can bring both physical and psychological aesthetic experiences to people [4]. Traditionally, auditory art can only be experienced through the auditory senses, and the form of experience is relatively single.

With the development of multimedia technology, people put forward higher requirements for the aesthetic experience of auditory art and seek more diverse forms of artistic expression. Taking the theory of wisdom education as a guide and exploring improvement countermeasures based on reflecting on the problems in today's intelligent piano-assisted basic piano teaching in colleges and universities are both a deepening of the theory of basic piano teaching and an application and deepening of the theory of wisdom education in music education [5]. This study helps to clarify the status of intelligent piano-assisted basic piano teaching in colleges and universities. Although digital piano classes in colleges and universities have been offered for many years, there are still various problems and confusions in the actual teaching for various reasons. To reflect the advantages of this new combined teaching mode, take piano solo teaching as an example, and use the traditional piano individual lessons for teaching arrangements. This research study will reflect on these problems and their causes, using the theory of wisdom education, which will help the healthy development of intelligent piano-assisted basic piano teaching. The exploration of countermeasures for intelligent piano-assisted basic piano teaching also helps to improve the efficiency of intelligent piano-assisted basic piano teaching at the same time [6]. The visualization of sound provides people with a new form of visual and auditory expression for the appreciation of auditory art. The new visual interpretation of auditory art adds to the expressiveness of auditory art, providing people with the dual beauty of the combination of visual and auditory senses, and the visualization of sound is conducive to people's deep understanding of the formal structure and emotional content of artworks. For example, in the live performance of the Czech Philharmonic Orchestra, the designer graphically designed the performance rhythmically and displayed it in 3D synchronously with the performance, bringing a visual feast to the audience.

This paper has an in-depth study of piano timbre. Through the piano pronunciation principle and the actual piano signal, the factors that affect the piano timbre are deeply studied. In addition, various existing audio analysis and synthesis techniques have been studied in depth. This paper studies common pitch recognition algorithms and fast pitch recognition algorithms for computer processing. The piano tone is reconstructed through the basic model of the piano mono signal. According to the factors that affect piano timbre, a timbre synthesis model with timbre parameters and editable timbre is designed. We design a synthesis method based on the timbre synthesis model, which can meet the requirements of timbre accuracy and real-time timbre synthesis by computer. We carried out the software and hardware design and equipment selection of the piano sound library generation system. In addition, the language used for software system development is C#. The system can realize the functions of piano audio acquisition, timbre editing, and timbre synthesis. It is experimentally verified that the timbre synthesized by using the existing timbre model is not realistic enough and has the characteristics of obvious electronic synthesized timbre, which cannot meet the requirements of digital piano and electric piano for high-quality timbre. By establishing an audio synthesis model, timbre editing can be realized, and the timbre can be synthesized to meet the requirements of piano sound realism. If the agent chooses from these actions, this action is called developing what is currently known about the value of the action. Conversely, if the agent chooses nongreedy actions, it is called temptation because it can improve the estimation of the value of nongreedy actions. Define appropriate timbre parameters to meet the dual requirements of timbre modification and timbre realism. Design a timbre synthesis method that enables rapid computer synthesis of timbres in real time while satisfying timbre realism and high accuracy. Design and implement a piano timbre library generation system to meet the common audio processing and timbre library synthesis functions. Several existing timbre synthesis models are introduced and simulated, but the synthesized timbres are not ideal; an audio synthesis model with editable timbres is proposed and the timbre synthesis steps are introduced; experiments show that timbre modification can be achieved by editing the timbre parameters of this model, and at the same time, the synthesized timbres meet the requirements of authenticity and high fidelity of piano timbre synthesis.

### 1.1. Status of Research

Pitch, timbre, intensity, and length are the four most important basic characteristics of music, and they lay the foundation for the entire piece of music. Pitch indicates the frequency of vibration of a sound; timbre refers to the different characteristics of a sound and represents different instruments; intensity, also known as loudness, indicates the size or strength of a sound; and length refers to the duration of a note from its beginning to its end [7]. In addition to the basic characteristics, there are complex characteristics like intervals, melody, rhythm, and overall characteristics like style, emotion, and category. Music features are an important basis for the study of music using computer technology, and the extraction of music features is an essential step for research in areas such as music retrieval and music signal processing. Music has many storage formats, and MIDI is a structured music data format that is widely used in research in the field of computer technology [8]. The total score of the work is excellent in the range of 90–100, good in the range of 80–89, average in the range of 70–79, qualified in the range of 60–69, and effort is needed in the range of 0–59. The design of piano fingering is closely related to the musical notes and piano keys, and the note information includes information such as pitch, pitch length, and pitch intensity, among which the pitch is associated with the piano keys; therefore, the pitch will be an important musical feature for the automatic generation of piano fingering research in this paper [9]. As piano fingering is closely related to the key position of the fingers, and the key position information represents the pitch information of the notes in the concrete form of piano playing, if we know the key position of each note on the piano keyboard, then we can follow the fingering rules to complete the performance of the music [10]. Therefore, this paper will directly use the MIDI file of the music and extract the key information contained in it as the musical characteristics.

The positions and forms of hands and fingers are represented as HMM states, and the resulting sequences of executed notes are modelled as emissions associated with HMM transitions, and a search is performed using the Viterbi algorithm to find the most likely sequence of state transitions [11]. Zioga et al. proposed an HMM-based piano fingering annotation method for both hands that can handle both single notes as well as chords [12]. In most of these results, machine learning is used to automatically generate piano fingering sequences based on hidden Markov models, since piano fingering sequences are to be generated based on the note sequences of music [13]. The rule-based knowledge base system approach mainly refers to music composition relying on symbolic operations and rule constraints. The advantage of this type of approach is that it has accurate logical reasoning and every act made by the system can be interpreted. A genetic evolutionary algorithm is a method that imitates the process of the genetic evolution of species in biology, first constructing a suitable adaptation function, and then deciding the evolutionary direction of candidate chromosomes through the adaptation function to perform global optimization, which is considered a very effective method in the field of music generation [14]. Being able to imitate production works and even make innovative works proves the applicability of the design. The questionnaire was used to collect frontline teachers' attitudes and opinions on the design of interactive learning resources and the learning effects of students using interactive learning resources. Most teachers gave positive evaluations to the design and production of interactive learning resources. The main problems of neural network-based music generation are as follows: the main problem of neural network-based music generation is that the music features are more complex and the training method is too single, so the quality of the generated music is not guaranteed. However, the neural network-based music generation method has less human involvement and does not require professional knowledge of music theory, and once the model is trained, the generation speed is fast and music can be generated [15]. Therefore, in this paper, after studying the existing music generation techniques, we choose the neural network-based music generation method.

To make the model fully consider the real music composition process, this paper reviewed the information about music composition and interviewed music composers about the specific process of composing music. After further research, these abstract conceptual constraints are converted into computer language, and a chord progression reward mechanism and a music theory rule reward mechanism are constructed and introduced into the critic network constructed in this paper.

## 2. Analysis of Piano Tone Teaching in the Context of Artificial Intelligence Interaction

### 2.1. Artificial Intelligence Interaction Background Design

**Figure 1:**
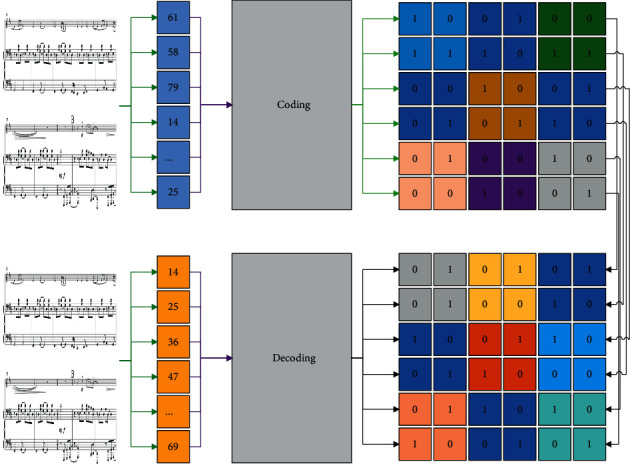
Generating a music framework.

There are two directions in the music generation problem, monophonic music generation, and multitrack music generation. There is no good solution so far on how to represent the connection between different tracks with mathematical models in multitrack music generation, and multitrack music generation requires more stringent training data compared to monotrack. The existence of these problems causes the music tracks generated by multitrack music algorithms to be confusing, of low quality, and lacking in artistic aesthetics; therefore, the music generation in this paper is mainly aimed at monotrack music generation [16]. There are many fields involved in deep learning and many types of network models have emerged during the development process, convolutional neural networks (CNNs), RNNs, LSTMs, etc. Among the abovementioned network types, CNN is mainly used in the field of image processing to extract feature information in images utilizing convolutional kernel, and it can build multi-layer convolution to extract higher dimensional information features, which is beneficial to image recognition and classification. Hoch Reiter and Schmid Huber proposed the LSTM network framework, whose structure is shown in Figure 1. RNN adds three gates, namely, a forget gate, an input gate, and an output gate. Through the gate to control the information, and protect and control the state of the cells, the effect of learning long-term dependence on information is achieved.

In the implementation of interactive sound visualization, what determines the final visual representation of sound is the transformation patterns and association rules between sound and visual information, and the final visual representation presents a wide variety of variability due to different transformation patterns and rules. Conducive to image recognition and classification. RNN is mostly used in the field of natural language processing. Unlike the feedforward neural network, RNN introduces a self-loop structure, which is characterized by being able to deal with issues related to the input information. Randy and Ben define this way of transforming sound information to visual information through some attribute of association rules as “mapping,” where different modes of transformation and association rules are different mappings. The term “mapping” is originally a mathematical term that refers to the unique correspondence between elements in a set of two elements [17]. In the mapping of sound visualization, sound information and visual information form two source bases for information matching, and some association rules are used to correspond to the information in the source bases and finally realize the transmission and transformation between audiovisual information.

It is different from mathematical mapping, and the “mapping” in this paper emphasizes some overall connection and transformation rules between audiovisual information from the macroscopic aspect, rather than referring to the unique correspondence of each element in it.

(1) $$\begin{array}{c} f_{t}=\sigma \left(W_{f}\cdot \left[h_{t},X_{t}\right]-b_{f}\right). \end{array}$$

The adaptation of learning resources to learners is manifested by the fact that learners can choose the learning content and the presentation of the learning content according to their own needs. The adaptation of learning resources to learners is manifested by giving immediate response and intelligent processing according to the dynamic information generated in the learning process of learners, such as providing different learning contents for learners according to the different answer situations of learners; recording students' learning; simulating problem situations in life, etc. And finally. realize the transmission and conversion between audiovisual information, which is not completely equivalent to mathematical mapping. The “mapping” in this article emphasizes a certain overall connection and conversion rule between audiovisual information from a macro perspective. Therefore, the support of learning resources for operational interaction can be summarized in five aspects: learner control, adaptivity, convenience, learning to monitor, and contextuality.

(2) $$\begin{array}{c} i_{t}=\sigma \left(W_{i}\cdot \left[h_{t},X_{t-1}\right]-b_{i}\right), \end{array}$$

(3) $$\begin{array}{c} o_{t}=\sigma \left(W_{o}\cdot \left[h_{t-1},X_{t-1}\right]+b_{o}\right), \end{array}$$

(4) $$\begin{array}{c} C_{t}=\coth\left(W_{c}\cdot \left[h_{t-1},X_{t-1}\right]-b_{c}\right). \end{array}$$

This stage is not an isolated existence, from aesthetic intuition to aesthetic understanding can produce relevant emotional and affective experience, it is the central outlet of various psychological content and form network structure in the aesthetic process, throughout the entire aesthetic process. When people are assimilated by the content and melody of sound work, they will integrate their body and mind into the sound art and produce the corresponding emotional experience, as in Gestalt psychology's mind-object isomorphism. This emotional experience unfolds along with the perception and understanding of the work and is an involuntary and intense psychological experience, which will be retained in the aesthetic memory for a long time even after the aesthetic process is over.

**Figure 2:**
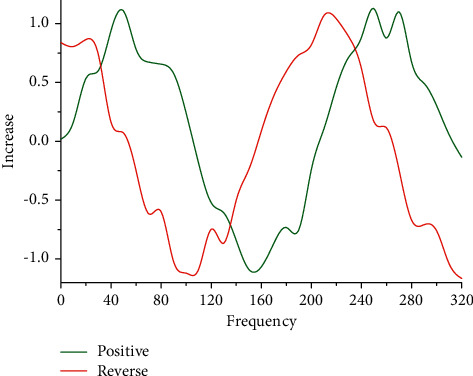
Sonogram.

Sound is a type of wave and therefore has the common properties of waves. Frequency, amplitude, and period are three important physical quantities that can describe the properties of sound waves. Frequency is the number of periodic regular changes per second, and for the frequency of sound, it is the number of sound waves that produce periodic vibrations in a second from the source. Amplitude is the maximum of the range of vibrations that a vibrating physical quantity can reach, and the amplitude of a sound determines the size of the sound. Sound waves exhibit periodic changes in their vibrational state during propagation, which describes the arrangement of individual repeated pressure changes of sound waves. The motion of the molecules of the medium is not visible to people, but according to the process of physical sound generation and physical properties, the conversion of the audiovisual mapping of physical sound can be achieved in three directions. Sound waves can be represented numerically and precisely by physical quantities such as frequency and amplitude, and through the perception of the extremely physical properties of the physical sound generation process, sound waves in physics are usually described and calculated mathematically in terms of the commonality of their generation principles, that is, a curved sonogram describing the periodic changes of sound energy at different moments experiencing different displacements utilizing mathematical number axes, as shown in Figure 2. The sonogram can accurately show the numerical variation of physical quantities of sound waves such as frequency and amplitude with time, which is important for the analysis of sound waves.

According to the physical nature of sound waves, a mapping transformation can be performed utilizing visual description, at which point the sound map becomes another form of sound existence, a visual feature of physical sound in human perception of sound, an external representation of sound that is based on objective knowledge of the physical characteristics of sound with unique certainty. Professional sound processing and editing software are based on this form of audiovisual conversion.

(5) $$\begin{array}{c} L\left(\theta_{M}\right)=\frac{1}{I}\overset{I}{\underset{i=1}{\sum }}\left(Y\;\ln\;Y-\left(1+Y\right)\ln\left(1+Y\right)\right). \end{array}$$

Software table technology uses algorithms to replace the hardware timbre library on the sound card and playback MIDI instrument sounds via CPU calculations in real time. The advantages of software table technology are that it produces high-quality sounds, is inexpensive, and is easily upgradeable [18]. Corresponding emotional experience is generated, such as the same shape of mind and object in Gestalt psychology, and psychological emotions will be generated with physical perception. However, the software table synthesis algorithms of different companies in the market are all trade secrets and cannot be obtained for free, and the synthesis results of software table synthesis algorithms vary from company to company, and their levels vary greatly. Therefore, an in-depth analysis of tone synthesis algorithms is needed.

(6) $$\begin{array}{c} P\left[S_{t+1}|S_{t}\right]=PP\left[S_{t+1}|S_{1},......,S_{1}\right]. \end{array}$$

There is a reason for the creation of new things, and humankind has never stopped searching and innovating in the field of art. Studies of the piano's development have shown that it is a product of the fusion of music and science and that it has become progressively more robust because of the improvement of science and technology. The piano is a keyboard instrument that produces sound by striking the strings through the power of the wrist, hand, and fingertips, so it also belongs to the category of percussion instruments, combining the attributes of a harmonious and dazzling instrument and a percussion instrument. But whatever changes this instrument underwent as it moved forward, it ultimately had a purpose around it, one to fill in the scraps of the age for the aural world with the finest sounds or combinations of different kinds of sounds, from which it gradually changed into aesthetic satisfaction.

(7) $$\begin{array}{c} \text{Notes Accuracy}=\frac{\sum_{m=1}^{M}\sum_{i=1}^{N}p\left(y_{im},y_{im}^{2}\right)}{M+N}. \end{array}$$

Human language as instruction has two different forms of material bearing, namely, speech and writing. Speech belongs to the formal expression of language in the human body, and writing belongs to the symbolic form of the external presentation of language. The two belong to the expression of language in different spatial dimensions. Under the structured system of human linguistic knowledge, speech and text correspond to each other, and text is the visual representation of speech. Human beings have a special function of converting speech and words in real time and accurately, which is done according to human speech perception. Pure Tone collects the high-quality timbres of 9 famous European pianos, up to 7 layers of timbre performance, and 128 realistic timbres. The reading centre in the brain is responsible for linking the visual and auditory symbols of words according to the intentional requirements of the words, thus allowing the person to establish the audiovisual link of words, i.e., to hear the pronunciation and write the words. Usually, such recognition is uniquely deterministic within the same linguistic structural system. The audiovisual transformation between the two bearer forms of language is based on the accepted standard of decoding instructions for human linguistic knowledge, has a broad agreement in human society, and produces a visual commonality in people.

### 2.2. Experimental Analysis of Piano Tone Teaching

**Figure 3:**
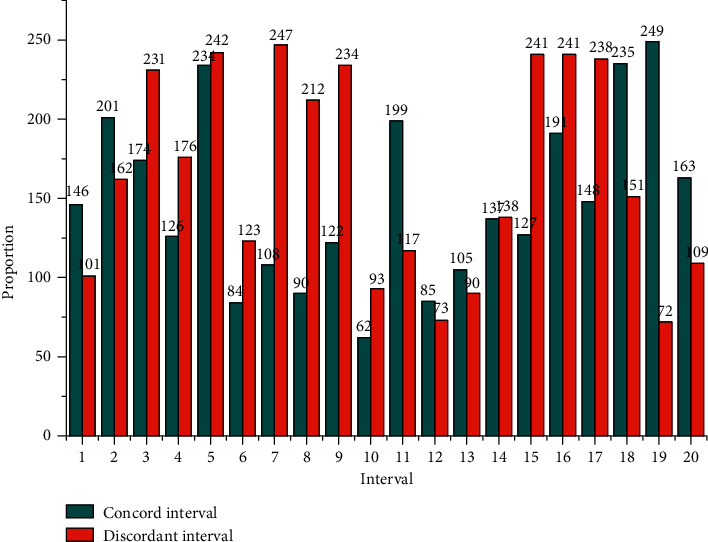
Interval concordance relationship.

In the timbre composition analysis, we have concluded that all frequency components in the frequency domain work together to form a full and rich piano tone, and that overtone frequency components alone cannot be used for piano tone synthesis. The next step is to investigate the different effects of different frequency components on the formation of piano timbre [19]. In piano timbre, the fundamental and its overtone series play a major role in forming the timbre, and the timbre synthesized using only this frequency component can simulate the main timbral qualities of the piano. The frequencies of the telharmonic play an essential role in forming a full and rich piano tone, and the more interharmonic frequencies used in synthesis, the closer the synthesized tone is to the original piano tone. The part of the frequency spectrum whose frequencies are below the fundamental frequency forms the sound of a piano key struck now it is pressed. The intervals are divided into concordant and discordant intervals according to the auditory perception that the harmonic intervals give to the human ear. Every art form can bring people a dual aesthetic experience of physical and psychological. In the traditional sense, auditory art can only be experienced through auditory senses, and the form of experience is relatively simple. The interval's harmony is a characteristic that reflects the basic nature of the interval and is determined by the ratio of the vibrational frequencies of the two tones forming the interval. An interval that sounds pleasant and blends are called a concordant interval, while an interval that sounds harsher and does not blend is called a discordant interval. Consonant intervals include very fully consonant intervals, fully consonant intervals, and imperfectly consonant intervals. The relationship between the degree of interval harmony is shown in Figure 3.

From Figure 3, the time-domain waveforms of the three synthesized timbres differed among the three sets of experimental parameters, indicating that the parameters of the synthesis model can effectively change the time domain waveforms of the timbres. At the same time, the time-domain waveforms of the synthesized timbres are very similar in terms of the general characteristics of the waveform changes, and they all satisfy the three phases of piano articulation: onset, decay, and fade [20]. The frequency-domain waveform plots of the three synthesized timbres are different for the three sets of experimental parameters, indicating that the parameters of the synthesis model can effectively change the frequency domain waveforms of the timbres. Sound visualization provides a new form of expression that combines audiovisual art for people to appreciate auditory art. The new visual interpretation of auditory art adds the expressive power of auditory art, provides people with the dual beauty of audiovisual sensory, and visualizes sound. Performance is conducive to people's deep understanding of the form, structure, and content emotions of artistic works. At the same time, the frequency-domain waveforms of the three timbres have the same characteristics: the frequency distribution is clean and clear, the frequency amplitude is larger at the octave, and the rest of the frequency amplitude is smaller, and there are more frequency components with smaller amplitude at the lower frequency band, which form the percussive sound of the piano now of pressing. Therefore, it can be judged that it belongs to the same category of piano tone.

The synthesized model timbre parameters can be set to change the time domain waveform and frequency domain waveform of the timbre and combined with the factors affecting the timbre, the timbre parameters must be changed to change the synthesized timbre; at the same time, the synthesized timbre retains the characteristics of the piano timbre in the time domain and frequency domain, in the time domain: it has three phases: onset, decay, and fade, and the sound rapidly decreases in the waveform and then continues to oscillate; in the frequency domain: the frequency distribution is clean. In the frequency domain, the frequency distribution is clean and clear, with larger frequency amplitude at the octave and smaller amplitude at the rest of the frequencies, and more frequency components of smaller amplitude at the low-frequency band that form the piano percussion. The synthesized timbre is a piano timbre, which satisfies the requirements of the piano timbre library generation system for timbre authenticity and high fidelity. Tone editing experiments can prove that using the audio synthesis touch pattern of editable tones in this paper for tone synthesis can change the synthesized tones by adjusting the tone parameters, and at the same time can meet the high-fidelity requirements of the piano tone generation system.

**Figure 4:**
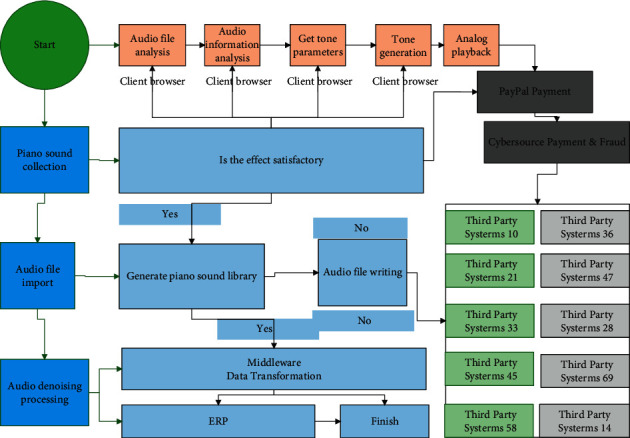
Flowchart of tone library generation.

The main steps of piano tone library generation using tone library generation software include: source input, parameter editing, tone library generation, and the software displays audio/tone information and supports simulated playback of synthesized tones to assist the user in generating satisfactory tones and tone libraries. Explore the basic model of piano monophonic signal, reconstruct piano timbre through the basic model; design timbre parameters and an audio synthesis model of editable timbre according to the factors that affect piano timbre. The flowchart of tone library generation is shown in Figure 4.

The software supports two types of audio input methods: piano sound acquisition and import of existing audio files, which requires denoising of the audio after piano sound acquisition and audio file parsing to obtain audio data information. Then, the audio data spectrally transformed and the timbre characteristics are analysed; the timbre parameters are obtained through the human-computer interaction interface and the piano timbre is generated. If you are satisfied with the sound effect, you can save it as an audio file or directly generate and save the piano sound library in batch; if you are not satisfied with the sound effect, you can re-edit the sound parameters through the HMI to generate the sound.

With the combination of smart piano and traditional piano teaching, the teaching arrangement is divided into individual and group lessons crossed by piano solo teaching as an example. Six weeks as a systematic teaching cycle can be based on the intelligent feedback of the intelligent piano and the feedback of the manual teacher as experimental data, to do a summary analysis of the course progress, quality. The first and second weeks are in the form of group lessons on smart piano, the third and fourth weeks are in the form of individual lessons of smart piano, and the fifth and sixth weeks are in the form of individual lessons of a traditional piano. Taking the example of “Chopin's Narrative in G minor” as a case study, we started teaching from the first week to the sixth week, based on the combination of students' receptiveness, basic piano ability, and the difficulty level of “Chopin's Narrative in G minor,” from the regular step-by-step teaching process the course is based on the combination of students' receptiveness, basic piano ability, and the difficulty level of the “Chopin's G minor.” Each week, student's feedback is combined with the final assessment of student completion, thus demonstrating the advantages of this new combined teaching model. The system can realize the functions of piano audio collection, timbre editing, and timbre synthesis. It has been verified by experiments that the timbre synthesized by the existing timbre model is not real enough and has obvious characteristics of electronically synthesized timbre, which cannot meet the high-quality requirements of digital pianos and electric pianos. As an example, solo piano instruction is arranged in the form of a single traditional individual piano lesson [21]. The six weeks are used as a systematic teaching cycle, and the weekly lessons are summarized and analysed based on the combination of manual teacher feedback and intelligent feedback from the smart piano in the sixth week as experimental tracking data. The first six weeks were spent in the form of traditional individual piano lessons. Taking the “Chopin narrative in G minor” as a case study, the first week to the sixth week, based on the combination of students' receptiveness, basic piano ability, and the difficulty of the “Chopin narrative in G minor”, each week combined with the data obtained from students' feedback, and finally checked students' completion. The final check of the students' completion is based on the data obtained from their feedback each week.

The two cases of the six weeks were compared in terms of different lesson formats and teaching methods, and the comparison shows that the combination of smart piano and traditional piano teaching is feasible and replicable. It is possible to try to incorporate “smart piano” into the existing piano teaching. Piano lessons with the “smart piano” application use both individual and group lessons, which are no longer mechanical and monotonous when combined with the traditional individual lessons. This new form of teaching is mainly based on intelligent technology, which can use pictures and audio to teach the students what they need to teach in oral and written form. Compared with the traditional teaching model, this is more stringent for students' learning quality, widens the path of students' knowledge acquisition, and improves teaching efficiency and quality.

## 3. Results and Analysis

### 3.1. Artificial Intelligence Interaction Performance Results

During the process of reinforcement learning, the intelligence will continuously estimate the value of states and actions, which is the basis for making action selection. When training reaches a mature stage, then the intelligence will mostly choose the action with the highest estimated value to perform, which will maximize the total gain and achieve our goal. However, at the beginning of training, the intelligence does not know which actions are really “good actions,” so the problem of how to make action selection is called the action selection mechanism. The intelligence continuously estimates the value of an action so that at any given moment there is at least one action with the highest estimated value, and these actions are called greedy actions. If the intelligence chooses from these actions, this behaviour is called exploiting (exploit) the currently known knowledge about the value of the action. Conversely, if the intelligence chooses a nongreedy action, it is said to explore, because this improves the estimation of the value of the nongreedy action. “Exploitation” is correct for maximizing the desired benefit in the current moment, but “exploration” may maximize the overall benefit overall. And in an active choice, exploitation and probing cannot be performed simultaneously.

**Figure 5:**
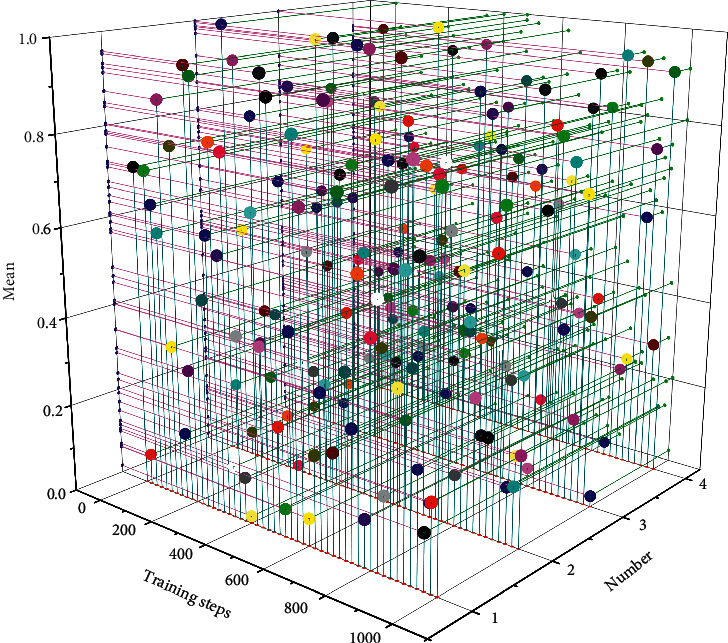
Comparison of average yield across music segments.

The average gain rate comparison statistics were obtained from the piano fingering generation experiments based on the constructed Q learning network using the key sequences of three music clips respectively, as shown in Figure 5. The average gain rate is the average of the ratio of the total gain to the optimal gain of a training ground, which reflects the superiority of the learning effect and the goodness of the result of the intelligence averaged over a certain range of training steps.

From Figure 5, when the number of training steps is relatively small, the average gain rate of each music fragment is unstable, the results are not satisfactory and fluctuate greatly, and as the number of training steps increases, the bits of intelligence learn to acquire better and better fingering rules, and the total gain gradually increases. The average gain rate stabilized earlier and later due to the different number of keys in each segment, and the segments with more keys require a larger number of training steps to ensure a better experimental result. The fingering sequences with larger yields were selected as the final output fingering sequence results in the training step interval where the average yield of each music fragment stabilized, respectively.

**Figure 6:**
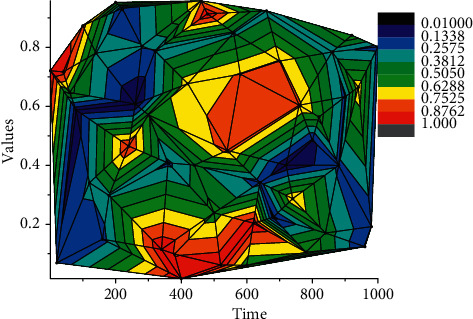
Plot of generated music versus sample music and difference sound spectrum.

In terms of the cumulative score trend, the machine-generated fingering may be slightly lower than the professional fingering in the intermediate steps, but in general, the scoring trend of the machine-generated fingering for the three music clips is consistent with the professional fingering; in terms of the score rate trend, the scoring rate of the machine-generated fingering for clip 1 and clip 2 is consistent with the professional fingering, and the scoring rate of the machine-generated fingering for clip 3 shows some fluctuations. This indicates that there is still some room for improving the stability of the generated fingering results for music fragments with many notes, but for music fragments with a small number of keys, it can generate high-quality, stable fingering, as shown in Figure 6.

After the above experiments, the character-level music generation network Melodists constructed in this paper can accomplish the end-to-end music generation task and can learn the dependencies of notes on the time scale from the training data, but the Melodists network only uses pure LSTM units and the structure is too simple that it cannot learn such complex data as music well. In this paper, we analyse many generation results and find that the generated music often contains many duplicate fragments, the generated music lacks structure, does not follow the rules of music theory, and lacks artistic aesthetics, and we also find that these problems exist in existing music algorithms. Therefore, this paper improves on the Melodists network constructed above and introduces a reinforcement learning algorithm to solve the above problems by combining deep learning and reinforcement learning.

### 3.2. Results of Teaching Piano Tone

**Figure 7:**
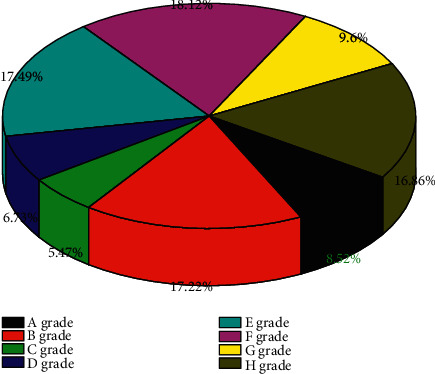
Thinking maps evaluation results statistics.

To test whether students achieve a deeper level of conceptual interaction when using interactive learning resources for learning, the teacher should evaluate the mind maps drawn by students during the course, referring to the evaluation criteria for scholarly mind maps in terms of whether the mind maps meet the cognitive level of the students, the appropriateness of the choice of keywords, the connection of relationships between key objects, the presence of scientific errors, aesthetics, etc. The statistics of the evaluation levels of the student-drawn mind maps were obtained as shown in Figure 7.

Through the above evaluation of the students' mind maps, most of the students' mind maps were able to reach the level of C and above, and the mind maps were in line with the student's cognitive level, with appropriate keywords, accurate hierarchical relationships, and no scientific errors, but the aesthetic aspects of the mind maps were still lacking. Students were able to make correct connections between the new content learned in this lesson and the mind maps are drawn in the previous lesson, clarify the hierarchical relationships between concepts, and make correct connections between the new content and the content they had already learned in their minds in the previous lesson.

**Figure 8:**
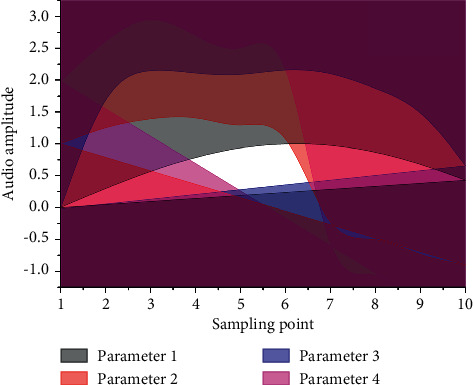
Comparison of the time-domain waveforms of the synthesized tones of the four sets of experimental parameters.

To test the effectiveness of students' learning using the interactive learning resources, and intelligent design work evaluation gauge is designed to evaluate the completion of students' classroom exercises in each lesson. Moreover, the multitrack music generation requires more stringent training data than the single-track music. The existence of these problems causes the confusion, low quality, and lack of artistic aesthetics of the music tracks generated by the multitrack music algorithm. The evaluation gauge evaluates the work in three dimensions: hardware assembly, pictorial programming, and work completion, with ten dimensions and a score of 10 points for each dimension. The total score of the work is excellent in the range of 90–100, good in the range of 80–89, fair in the range of 70–79, pass in the range of 60–69, and needs work in the range of 0–59. The work of 10 students within the AI club was evaluated according to the scale, resulting in the student work evaluation grade statistics shown in Figure 8.

The Smart Design Work Evaluation Scale evaluates students' classroom practice work, and the applicability of the design is demonstrated by the fact that students met the learning objectives after learning with the interactive learning resource and were able to imitate and produce work or even make innovative work. The questionnaire collected frontline teachers' attitudes and opinions about the design of the interactive learning resources and the learning effects of students' use of the interactive learning resources. Most of the teachers gave positive comments on the design and production of the interactive learning resources and believed that the use of the interactive learning resources could guide students to actively control their learning progress, independently assess their learning status, promote the independent construction of learners' knowledge, and realize the deep active interaction between learners. The use of interactive learning resources can guide students to actively control their learning progress, assess their learning status, promote the independent construction of learners' knowledge, and achieve deep active interaction between learners and learning resources.

Most surveyors say that smart pianos mostly used in group lessons and the equipment is old, and students rarely use smart pianos in their daily piano training. In the scenario of basic piano teaching, smart pianos are useful for technique teaching and psychological teaching; in the more complex and high-end teaching scenario involving performance teaching, smart pianos are not suitable for this purpose. In the current educational environment, the use of intelligent pianos is still very limited. In the current situation of intelligent pianos to assist in basic piano teaching, the author first analyses the causes of the problem from the results of the survey and the literature, including the pressure of teachers to research, the uneven level of students, the lack of professional orientation for students, the lack of contact with intelligent piano courses and the lack of continuity in learning, and the lack of investment in hardware in schools.

## 4. Conclusion

In this paper, an audio synthesis model with editable timbre is proposed, in which the timbre parameters: maximum octave, amplitude modification coefficient, synthesis parameters, and modification type are set to realize the editable timbre. The synthesis and editing experiments show that the timbre synthesized by this model can meet the high-fidelity requirements of the piano timbre generation system. The model can be used to synthesize timbres to meet the flexibility and authenticity requirements of the piano timbre generation system. The existing piano timbre synthesis model is analysed and experimented with, and the difference between the synthesized timbre and the actual piano timbre is very large, which cannot meet the high-fidelity requirement of the sound source. In this paper, based on the multiplicative harmonic model of monophonic signal, combined with the variability of timbre characteristics, an audio synthesis model with editable timbre is proposed, and the editable timbre is realized by modifying each timbre parameter of the model. The experimental results show that the piano timbre synthesized by this model can meet the system's requirements for flexibility and realism of timbre library generation. The system software includes audio file reading and writing touch block, audio information analysis module, timbre parameter acquisition module, timbre synthesis module, and simulated performance module. Compared with the traditional methods, the use of this system to generate piano timbre libraries is inexpensive and simple to operate, and it meets the demand for authenticity and personalization of sound sources for digital pianos and smart pianos.

## Data Availability

The data used to support the findings of this study are available from the corresponding author upon request.
